# Bayesian Estimation of Three-Dimensional Chromosomal Structure from Single-Cell Hi-C Data

**DOI:** 10.1089/cmb.2019.0100

**Published:** 2019-11-07

**Authors:** Michael Rosenthal, Darshan Bryner, Fred Huffer, Shane Evans, Anuj Srivastava, Nicola Neretti

**Affiliations:** ^1^ Science and Technology Department, Naval Surface Warfare Center, Panama City Division, Panama City, Florida.; ^2^ Department of Statistics, Florida State University, Tallahassee, Florida.; ^3^ Center for Computational Molecular Biology, Brown University, Providence, Rhode Island.; ^4^ Department of Molecular Biology, Cell Biology, and Biochemistry, Brown University, Providence, Rhode Island.

**Keywords:** Bayesian estimation, chromosome 3D structure, Hi-C

## Abstract

**The problem of three-dimensional (3D) chromosome structure inference from Hi-C data sets is important and challenging. While bulk Hi-C data sets contain contact information derived from millions of cells and can capture major structural features shared by the majority of cells in the sample, they do not provide information about local variability between cells. Single-cell Hi-C can overcome this problem, but contact matrices are generally very sparse, making structural inference more problematic. We have developed a Bayesian multiscale approach, named Structural Inference via Multiscale Bayesian Approach, to infer 3D structures of chromosomes from single-cell Hi-C while including the bulk Hi-C data and some regularization terms as a prior. We study the landscape of solutions for each single-cell Hi-C data set as a function of prior strength and demonstrate clustering of solutions using data from the same cell.**

## 1. Introduction

The use of whole-genome conformation capture techniques (3C) such as Hi-C (Lieberman-Aiden et al., 2009) has revealed that the three-dimensional (3D) organization of the genome plays a key role in regulating fundamental cellular processes such as transcriptional regulation, cell cycle progression, and cellular differentiation (Lieberman-Aiden et al., 2009; Naumova et al., 2013; Dixon et al., 2015). These studies generate contact maps describing the probability of observing interactions between any two regions of the genome, which can be associated with distance matrices between pairs of genomic loci. Methods developed to infer the 3D structure of chromosomes from these contact maps typically rely either on optimization-based strategies to minimize the difference between the inferred structure and the distance matrix (Rousseau et al., 2011; Hu et al., 2013; Varoquaux et al., 2014; Zou et al., 2016; Park and Lin, 2016), or on probabilistic modeling to find the most likely structure(s) given the observed contact probabilities (Baù and Marti-Renom, 2012; Zhang et al., 2013; Lesne et al., 2014; Szałaj et al., 2016; Adhikari et al., 2016; Rieber and Mahony, 2017; Trieu and Cheng, 2017).

While Hi-C is typically collected on bulk samples containing millions of cells, it is not clear how much the organizational features present in these population data sets reflect the 3D organization of chromosomes in individual cells. For example, it is not guaranteed that all observed long-range contacts appear simultaneously in each cell (Tjong et al., 2016). Thanks to recent advances in Hi-C technology, we can now study long-range interactions at the single-cell level (Nagano et al., 2013; Flyamer et al., 2017; Ramani et al., 2017; Stevens et al., 2017; Nagano et al., 2017). Single-cell Hi-C has confirmed many organizational principles described in bulk experiments, but their interpretation is not straightforward. For example, it is not yet clear whether topologically associated domains are 3D structural units in individual cells or a population feature that emerges when many cells are aggregated in bulk Hi-C experiments (Nagano et al., 2013; Flyamer et al., 2017), although recent work in *Drosophila* points to the former (Szabo et al., 2018).

The primary difficulty in inferring 3D chromosome structures from single-cell Hi-C data is the sparseness of the contact maps. Currently available methods rely on inference of missing data (Paulsen et al., 2015) or on polymer models with the optimization based on Markov chain Monte Carlo (Carstens et al., 2016) or simulated annealing techniques (Nagano et al., 2013; Stevens et al., 2017; Nagano et al., 2017). However, recovery of potentially missing long-range interactions in the contact matrix relies exclusively on the information contained within individual single-cell matrices.

Here we present a solution using Structural Inference via Multiscale Bayesian Approach (SIMBA3D), which utilizes bulk Hi-C to aid in recovering the contribution of interactions potentially missed in single-cell Hi-C contact maps. Our strategy is similar in principle to the one used in Tjong et al. (2016), where bulk Hi-C is decomposed into an ensemble of single-cell 3D structures. We build a generalized Bayesian framework that utilizes penalties associated with folding constraints and a prior derived from bulk Hi-C samples to infer 3D chromosome structure in single cells. The SIMBA3D software is available at (https://github.com/nerettilab/SIMBA3D).

## 2. Methods

### 2.1. Proposed framework

The primary goal of the inference is to efficiently explore a vast space of potential chromosomal structures and seek optimal solutions using contact matrices and other contextual data. This requires constructing objective functions with desirable properties and developing scalable algorithms to reach interpretable conformations in times that are practical for large-scale computations. As stated above, the problem of estimating chromosomal structure from single-cell data is challenging because these data are very sparse and noisy. To reach more realistic solutions, we implement a Bayesian approach that supplements the single-cell data with the bulk data. This technique helps fill the missing parts with structures corresponding to the population of cells and additionally imposes certain penalties to improve the quality of estimated structures. The penalties are designed in particular to favor uniform placement of points on the estimated curve and to force the curve itself to be smoother.

Suppose that the genome is partitioned into *n* equally sized, disjoint segments, or bins. Let *C* be the *n* × *n* data matrix obtained from an Hi-C experiment. The *ij'*th entry of *C*, call it *c_ij_*, represents the number of observed interactions between segments *i* and *j*, and thus, *C* is naturally a symmetric matrix. Suppose further that another data matrix *C*′ is available to us and represents the collective results of prior Hi-C experiments. For example, in the case of a single-cell Hi-C matrix *C*, we could also have available to us bulk Hi-C data from the same type of cell, or, alternatively, *C*′ could be equal to the sum of several other single-cell Hi-C matrices. Let *x_i_*∈${ \mathbb{R}}$^3^ be the center of mass of the *i*th segment, and let *X*∈${ \mathbb{R}}$^*n*×3^ be the collection of all such *x_i_'*s. The problem at hand is twofold: (1) to estimate the structure *X* from the Hi-C data matrix, and (2) to quantify differences in structures obtained either from the same or different data matrices. In both the estimation and analysis stages, we consider a structure *X* to be equivalent modulo scale, translation, and rigid rotation/reflection.

We define a posterior energy function *E* on the space of potential curves, ${ \cal X} = { \mathbb{R}^{n \times 3}}$—each curve containing *n* points (or nodes) in ${ \mathbb{R}^3}$—and use a gradient-based approach to solve for an optimal solution

\begin{align*}
\hat X = \mathop {{ \rm{arg}} \,{ \rm{inf}}} \limits_{X \in { \cal X}} E ( X \vert C , C \prime , a , b , \lambda ) , \tag{1}
\end{align*}

where *C* = (*c_ij_*) and *C*′ = (${c \prime _{ij}}$) are the single-cell and bulk contact matrices, respectively, *λ* is a vector of weights, and *a* and *b* are predetermined model parameters (described later). Let $M = \sum \nolimits_{j > i} {{c_{ij}}}$ and $M \prime = \sum \nolimits_{j > i} {{{c \prime }_{ij}}}$, respectively. The energy function *E* has several terms, each contributing to a certain aspect of the estimated curve:

\begin{align*}
E ( X \vert C , C \prime , a , b , \lambda ) = \frac { 1 }  { M } g ( X \vert C , a , b ) + { \frac { { \lambda _3 } }  { M \prime } } g ( X \vert C \prime , a , b ) + { \lambda _1 } { h_1 } ( X ) + { \lambda _2 } { h_2 } ( X ) { \mkern 1mu } . \tag { 2 } 
\end{align*}

We discuss one by one these quantities that comprise *E*.

#### 2.1.1. Negative log-likelihood term

The first term *g*(*X* | *C*, *a*, *b*) in Equation (2) is the negative log-likelihood of the contact matrix *C* given a curve *X*. This term follows a Poisson model (Varoquaux et al., 2014) with *a*, *b* being predetermined model parameters, as follows. Varoquaux et al. (2014) link the *ij'*th interaction count with the *ij'*th pairwise distance via the following probability model:

\begin{align*}
{C_{ij}} \sim { \rm{Poisson}} ( b \vert \vert {x_i} - {x_j} \vert { \vert ^a} ) \tag{3}
\end{align*}

for *j* > *i*, with ∥·∥ being the standard Euclidean norm, and for scalars *a* < 0 and *b* > 0. Since *a* < 0, the expected number of interactions between segments *i* and *j* is larger when the segments are located closer together in space, and this expected number behaves according to a power law with power *a*. Varoquaux et al. (2014) derive the theoretically optimal value of *a* = −3 from principles of polymer physics. Furthermore, the parameter *b* acts as a scaling parameter.

The probability mass function for the Poisson random variable given in Equation (3) is given by the following:

\begin{align*}
P ( { C_ { ij } } = { c_ { ij } } \vert X , a , b ) = { \frac { { { ( b \vert \vert { x_i } - { x_j } \vert { \vert ^a } ) } ^ { { c_ { ij } } } } { e^ { - b \vert \vert { x_i } - { x_j } \vert { \vert ^a } } } }  { { c_ { ij } } ! } } \;.
\end{align*}

Thus, given an Hi-C matrix with independent entries, the log-likelihood function is written as follows:


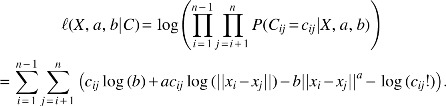


From the log-likelihood function above, one can see that for a given value of *a*, the parameter *b* is nonidentifiable because *ℓ*(*X*, *a*, *γ^a^b*|*C*) = *ℓ*(*γX*, *a*, *b*|*C*) for any scalar $\gamma > 0$. That is, changing *b* is equivalent to changing the scale of *X*, and since *X* is considered equivalent modulo scale, the choice of *b* is arbitrary. Define the function *g* as the negative log-likelihood function, dropping terms that are constant with respect to *X*, that is,

\begin{align*}
g ( X \vert C , a , b ) = - \mathop \sum \limits_{i = 1}^{n - 1} { \mathop \sum \limits_{j = i + 1}^n { ( a{c_{ij}} \log ( } } \vert \vert {x_i} - {x_j} \vert \vert ) - b \vert \vert {x_i} - {x_j} \vert { \vert ^a} ) \;. \tag{5}
\end{align*}

In structure estimation, we consider the parameters *a* and *b* to be fixed and known values; thus, we include them with the data *C* as given when writing the function *g*. The maximum likelihood estimate (MLE) of *X* is computed as the minimizer of *g*; that is,

\begin{align*}
\hat X = \mathop {{ \rm{arg}} \,{ \rm{min}} \,} \limits_{X \in { \mathbb{R}^{n \times 3}}} g ( X \vert C , a , b ). \tag{6}
\end{align*}

#### 2.1.2. Penalty terms

In situations when the data matrix *C* is sparse or noisy, the standard MLE in Equation (6) can be biologically unrealistic or even fail to converge. Thus, we design several additive penalty terms to regulate the maximum likelihood solution. The remaining terms in *E* as defined in Equation (2) can be viewed as imposing a prior distribution on the curve. These terms represent the prior belief that the curve displays some regularity. The term involving *h*_1_ penalizes variation in the distances between adjacent points on the estimated curves, while the term with *h*_2_ penalizes deviations from straightness. Since the weights *λ*_1_ and *λ*_2_, are typically small, these terms essentially discourage excessive variation in the distances between points and excessive bending of the curve. The second term in Equation (2) involves the negative log-likelihood of the bulk contact matrix *C*′ and represents the prior belief that the curve *X* will bear some resemblance to those found in the population. Together, these three terms drive the solution toward a smoother, more interpretable curve that conforms to both single-cell and bulk data.

These are constructed as follows.

1. **First Penalty**: Define the first penalty as


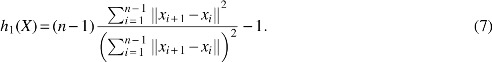


The interpretation of *h*_1_ is the following. Define $L ( X ) = \sum \nolimits_{i = 1}^{n - 1} \vert \vert {x_{i + 1}} - {x_i} \vert \vert$ as the length of *X*, and let ${ u_i } = { \frac { n - 1 }  { L ( X ) } } \vert \vert { x_ { i + 1 } } - { x_i } \vert \vert$ be the distance between the *i*th pair of adjacent points in *X*, when *X* has been rescaled to have length *n* – 1. Then, one can show that ${h_1} ( X ) = \sigma _u^2$, where $\sigma _u^2$ is the variance of the *u_i_'*s, and therefore, the effect of the penalty *h*_1_ is to reduce the variability of the distances between adjacent points of *X*. The configuration that minimizes *h*_1_(*X*) is such that all the *u_i_'*s are equal to 1, that is, adjacent points in *X* are all the same distance apart. The minimum value of *h*_1_ is 0 regardless of the value of *n*. Furthermore, notice that since *h*_1_(*γX*) = *h*_1_(*X*) for any *γ* > 0, *h*_1_(*X* + *y*) = *h*_1_(*X*) for any *y*∈${ \mathbb{R}}$^*p*^, and *h*_1_(*XR*) = *h*_1_(*X*) for any 3 × 3 orthogonal matrix *R*, the penalty *h*_1_ is invariant to scale, translation, and rotation/reflection.

2. **Second Penalty**: Define the second penalty as

\begin{align*}
 { h_2 } ( X ) = \frac { 1 }  { { n - 2 } } \mathop \sum \limits_ { i = 2 } ^ { n - 1 } { { \frac { ( { x_ { i - 1 } } - { x_i } ) \cdot ( { x_ { i + 1 } } - { x_i } ) }  { \vert \vert { x_ { i - 1 } } - { x_i } \vert \vert \vert \vert { x_ { i + 1 } } - { x_i } \vert \vert } } } . \tag { 8 } 
\end{align*}

The interpretation of *h*_2_ is the following. If *θ_i_* is the angle created by the triplet of points (*x_i_*_– 1_, *x_i_*, *x_i_*_+ 1_), and *y_i_* = cos(*θ_i_*), then ${h_2} ( X ) = \bar y$, the sample mean of all the *y_i_'*s. Therefore, the minimizer of *h*_2_ is such that cos(*θ_i_*) = −1 for all *i* = 2, …, *n* – 1. This occurs when *X* is a straight line with all *θ_i_* = *π*; hence, the effect of the penalty *h*_2_ is to enforce a level of smoothness to *X*. The penalty *h*_2_ has a minimum value of −1 regardless of the value of *n* and is invariant to scale, translation, and rotation/reflection.

3. **Bulk Prior**: The second term in Equation (2; the bulk prior) may be written as *λ*_3_*h*_3_(*X*) where we define

\begin{align*}
 { h_3 } ( X ) = \frac { 1 }  { { M \prime } } g ( X \vert C \prime , a , b ). \tag { 9 } 
\end{align*}

Notice that if we let $\tilde C = C + { \frac { { \lambda _3 } M }  { M \prime } } C \prime$ and $\tilde b = ( 1 + { \frac { { \lambda _3 } M }  { M \prime } } ) b$, then the sum *g*(*X* | *C*, *a*, *b*) / *M* + *λ*_3_*h*_3_(*X*) is equal to *g*(*X* | $\tilde C$, *a*, $\tilde b$) / *M*. Therefore, the effect of the penalty *h*_3_ is essentially to add a scalar multiple of *C*′ to the data *C* and perturb the parameter *b*. If *λ*_3_ is chosen to be small enough, then this penalty term will only slightly alter *C* and not overwhelm the original data with the bulk data. If *C* is sparse and *C*′ is not, then the addition of this penalty term with a small enough *λ*_3_ eliminates the sparseness of *C* by replacing many of the 0 entries with small numbers that are biologically more meaningful than random noise.

There are many other possibilities for penalty terms. However, for practical implementation of the optimization problem in Equation (1), we use only the penalties *h*_1_, *h*_2_, and *h*_3_ for three reasons. First, each penalty term has a straightforward and biologically meaningful interpretation. Second, the formula for each term is relatively simple and inexpensive to compute—in particular, since *h*_3_ becomes absorbed into the function *g*, the addition of this penalty requires an essentially zero increase in overall computation time. Third, along with the function *g*, we can write an analytical expression for the gradient of each penalty term, and therefore, we can write a gradient expression for *E*. By inputting the expression of ∇*E* to a numerical solver, we can maintain computational tractability on a personal computer for the large values of *n* typically seen in real data sets. Using the penalty functions, *E* can be written as

\begin{align*}
E ( X \vert C , C \prime , a , b , \lambda ) = \frac { 1 }  { M } g ( X \vert \tilde C , a , \tilde b ) + { \lambda _1 } { h_1 } ( X ) + { \lambda _2 } { h_2 } ( X ) \; , \tag { 10 } 
\end{align*}

where $\tilde C$ and $\tilde b$ are defined in the text following Equation (9), and we consider this objective function for the remainder of this work. The choice of the penalty weights *λ* is left to the user and can influence the solution greatly.

### 2.2. Multiscale gradient optimization for improved inference

The biggest challenge in solving the optimization problem given in Equation (1) comes from a nonconvex energy function and an extremely high-dimensional search space, which brings about multiple local optima and a tremendous computational cost. While the presence of the bulk and penalty terms helps to mitigate these issues by steering the search toward more realistic solutions, the computational complexity still remains a major hurdle.

#### 2.2.1. Gradient-based optimization

We take a multiscale, gradient-based approach, where the optimization at each iteration is performed using a gradient-based technique called Broyden–Fletcher–Goldfarb–Shanno (BFGS; Gill et al., 1981; Nocedal and Wright, 2006). Thus, it is helpful to derive an analytical expression for ∇*E* to a numerical optimizer. The gradient of *E* at *X* is written as

\begin{align*}
\nabla E ( X \vert C , C \prime , a , b , \lambda ) = \frac { 1 }  { M } \nabla g ( X \vert \tilde C , a , \tilde b ) + { \lambda _1 } \nabla { h_1 } ( X ) + { \lambda _2 } \nabla { h_2 } ( X ) \;; \tag { 11 } 
\end{align*}

therefore, to build the expression for ∇*E*, we need to compute the expressions for ∇*g*, ∇*h*_1_, and ∇*h*_2_, where *g*, *h*_1_, and *h*_2_ are defined in Equations (5), (7), and (8), respectively. The expressions for these gradients are presented in Supplementary Data.

#### 2.2.2. Multiscale optimization

**FIG. 1.**
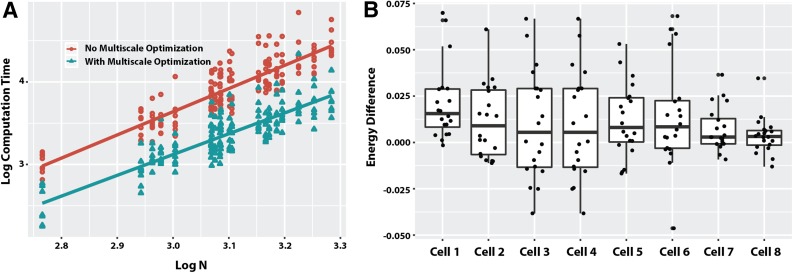
Improvement in computation time and final energy with multiscale optimization. Comparison of results, with and without the multiscale approach executed on all 20 chromosomes in each of the 8 cells, available in the mESC data set. **(A)** The log scale computation time is plotted over log scale number of nodes with regression lines fitted. **(B)** Boxplot of the difference in final energies obtained for each of the eight cells. The difference is computed by subtracting the energy obtained via the multiscale approach from the energy obtained without the multiscale approach. That is, a positive number here indicates that the multiscale approach yielded a lower energy solution.

To reduce the computation time to allow for a practical full-genome reconstruction, we implement a multiscale optimization technique. Compared with a standard approach that computes the full resolution optimization using a random initialization, the multiscale approach reduces computation time and limits the local solutions obtained. As shown in Figure 1B, this leads to solutions with smaller energies.

The multiscale optimization technique used in SIMBA3D is as follows. First, from a given full resolution contact matrix, we generate a series of new matrices decreasing in resolution, that is, decreasing in size, by recursively combining adjacent pairwise interaction counts to reflect a merging of adjacent genomic bins. One iteration of this process cuts the dimension of the contact matrix roughly in half. For each matrix generated in the series, we ignore the diagonal elements as we would in the original full contact matrix. Once we generate the multiscale series of matrices, we execute the series of optimizations in the reverse order, beginning with the smallest matrix and ending with the full matrix. We initialize the smallest optimization randomly from a standard multivariate normal distribution, obtain a solution, and then upsample this solution (i.e., interpolate between the solution nodes) to initialize the next larger optimization problem in the series. We continue this iterative process of solving successively larger optimizations, using an upsampled version of the current solution as an initialization to the next higher resolution problem, until we finish with the full solution. SIMBA3D implements this multiscale technique in Python, through which at each scale the optimization is solved using the BFGS method (Gill et al., 1981; Nocedal and Wright, 2006) with analytical gradient.

Although we solve several optimization problems in the above multiscale approach compared with just one in a standard approach, the computation time is significantly reduced. Since the smaller optimizations are relatively fast compared with the full resolution version, the multiscale approach is essentially a systematic way of cheaply providing a good initialization to the full problem. The combined cost of producing this initialization and executing the full resolution optimization is less than the cost of executing the full resolution optimization with a full resolution random initialization. Moreover, our experimental results show that we achieve on average a lower energy, that is, better quality solution using the multiscale approach compared with that of the standard approach of random full resolution initialization. An additional consequence of using the multiscale approach is that by design, the space of obtainable full resolution local solutions is limited by the initial smallest resolution. In extreme cases, the very small resolution problems may only have one solution; therefore, if one wishes to explore the local solution space of the full resolution by using different random initializations while still enjoying the benefits of reduced computation time, one must strike a reasonable compromise in initial scale size.

**FIG. 2.**
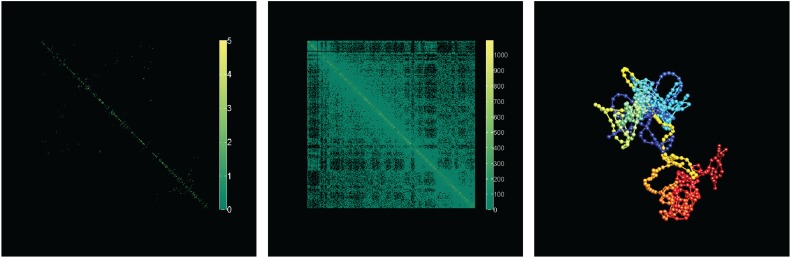
An illustration of chromosome structure estimation using SIMBA3D: the single-cell sparse contact matrix (left), the ensemble contact matrix (middle), and the final estimated structure (right).

Shown in Figure 2 is an example of an estimated structure obtained using SIMBA3D on chromosome 19 in the mouse embryonic stem cell (mESC) data set (Stevens et al., 2017). The left panel shows the single-cell contact matrix *C* (from cell 1) and the middle panel shows the ensemble matrix *C*′. The result of the estimation $\hat X$ is shown in the rightmost panel. The algorithm was applied for the parameter values *λ*_1_ = 0.5, *λ*_2_ = 1.0, and *λ*_3_ = 0.1.

## 3. Results

### 3.1. Simulated data

A complete estimation solution for a simulated configuration, intended as an illustration of the estimation process, is presented in Supplementary Figure S1. To verify the capabilities of SIMBA3D on more realistic data sets, we designed and executed a series of experiments on single-cell Hi-C data matrices that have been simulated from a known set of ground truth structures. The ground truth structure set {*X_k_*∈${ \mathbb{R}}$^100×3^, *k* = 1, …, *K*} was designed to exhibit cell-to-cell chromatin shape variability from a mean structure that was obtained from downsampling and smoothing an SIMBA3D solution from real Hi-C data, more extensively described in Section 3.2. For each *X_k_*, one can easily simulate a corresponding 100 × 100 single-cell Hi-C matrix *C_k_* using the Poisson model described in Equation (3). Any solution from SIMBA3D using *C_k_* can then be optimally scaled and aligned to the ground truth structure and then compared via the root mean square distance (RMSD) metric. With carefully designed experiments, one can make inferences about the solution quality that SIMBA3D produces under various circumstances using this RMSD to ground truth metric.

**FIG. 3.**
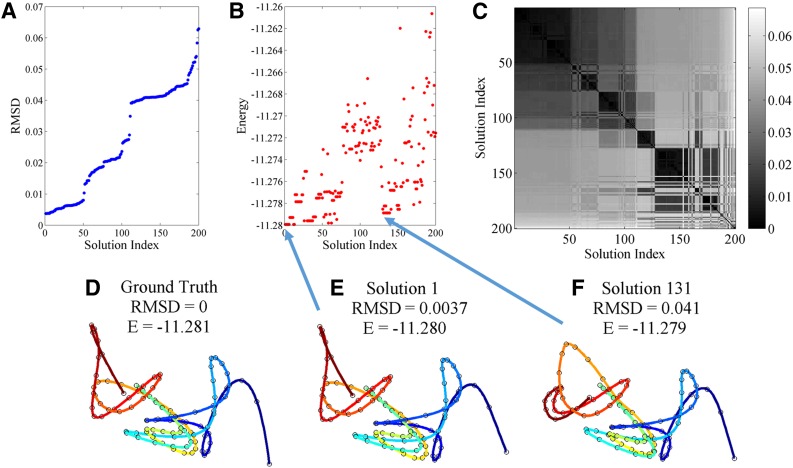
From one selected ground truth structure containing 100 nodes, we simulate a dense Hi-C matrix (not shown) from the Poisson model using *a* = −3 and *b* = 10^6^. We obtain 200 solutions from SIMBA3D using 200 random initializations and with penalty weights *λ*_1_ = *λ*_2_ = 0.001, and *λ*_3_ = 0. **(A)** A plot of RMSD to ground truth versus the solution index, where the solution index is ordered in ascending RMSD. Using the same solution set and index ordering, **(B)** a plot of the energy versus solution index. **(C)** The 200 × 200 pairwise RMSD matrix for each solution pair, also using the same index ordering as in **(A)** and **(B)**. **(D**, **E**, **F)** A 100-node structure with nodes connected via a colorized spline interpolated curve. The beginning node with index 1 is located at the blue end of the curve, and the ending node with index 100 is located at the red end of the curve. Also shown above each curve is its label, its RMSD to ground truth, and its energy value **(E)**. **(D)** The ground truth curve. **(E)** Solution 1, the solution with the lowest RMSD of the 200; and **(F)** Solution 131, the solution with the 131st lowest RMSD. Solution 1 is a near-perfect reconstruction with respect to ground truth, and Solution 131 is a similarly optimal solution with respect to energy, but exhibits a local reflection at the red portion of the structure, which leads to a much higher RMSD value. RMSD, root mean square distance; SIMBA3D, Structural Inference via Multiscale Bayesian Approach.

The first experiment is designed to verify that SIMBA3D can recover the ground truth structure exactly, up to a small error tolerance, when there are a sufficient amount of contact data, that is, when the data matrix is dense. From one selected ground truth structure, we simulate a dense Hi-C matrix from the Poisson model using *a* = −3 and a large value of *b* = 10^6^. Since in this situation there is ample contact data available to accurately reconstruct the curve, we use small penalty weights *λ*_1_ = *λ*_2_ = 0.001, and we set *λ*_3_ = 0 to forgo the unnecessary use of the population prior. Figure 3 analyzes the solution quality of 200 structure estimations obtained from SIMBA3D using 200 random initializations and without using the multiscale approach. Figure 1 shows that the 50 solutions with the lowest RMSD are essentially the same and recover the ground truth structure correctly up to a small error tolerance. However, due to the high dimensionality of the optimization problem, there are many other local solutions, for example, Solution 131, that are nearly globally optimal but exhibit a flipped or reflected portion of the structure. For this reason, RMSD and energy are only loosely correlated. The darker squares along the diagonal of the pairwise distance matrix in the upper right panel show evidence for the clustering of local solutions with respect to the RMSD metric.

**FIG. 4.**
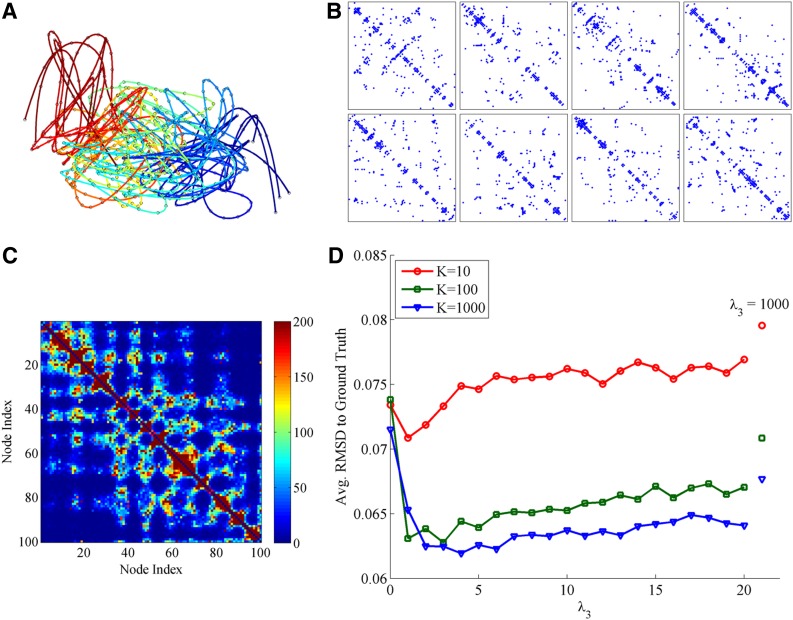
**(A)** The first eight ground truth structures in the simulated data set, all with the same scale, centroid, and mutual alignment over rigid body transformations. As with the structures shown in Figure 1, each structure has 100 nodes connected via a colorized spline interpolated curve. The beginning node with index 1 is located at the blue end of the curve, and the ending node with index 100 is located at the red end of the curve. **(B)** The nonzero elements of the simulated sparse Hi-C matrices *C_k_* for each respective ground truth curve *X_k_* in **(A)**. **(C)** The bulk data matrix that results from summing all *C_k_'*s together, excluding *C*_1_, for a data set of size *K* = 1000. **(D)** Plots RMSD versus *λ*_3_ averaged over 20 SIMBA3D solutions obtained for each of the first 8 simulated matrices (i.e., each data point represents the average RMSD to ground truth for 20 × 8 = 160 structures). The plot shows three curves corresponding to the scenario of using a data set of size *K* = 10, 100, and 1000 single-cell matrices.

The second experiment is designed to verify that incorporating the population prior in SIMBA3D when the single-cell Hi-C matrix is sparse improves the quality of the estimated solution. The experimental setup is the following. First, for each *X_k_*, *k* = 1, …, *K*, we simulated a sparse Hi-C matrix *C_k_* from the Poisson model using a value of *b* = 10. We then selected the first 8 matrices to perform the structure estimation with SIMBA3D, using several values of *λ*_3_, including *λ*_3_ = 0, and fixed values *λ*_1_ = *λ*_2_ = 0.1. When estimating the structure from matrix *C_k_*, the population prior makes use of the bulk matrix given by the sum of all matrices in the data set of size *K* excluding *C_k_*. For each value of *λ*_3_ and for each of the 8 chosen matrices, we obtain 20 solutions from SIMBA3D using 20 random initializations, again forgoing the use of the multiscale optimization feature in the software. Figure 4 plots the RMSD to ground truth value averaged over all 8 × 20 = 160 solutions for each value of *λ*_3_ tested. We repeat this experiment three times using *K* = 10, *K* = 100, and then the full *K* = 1000 structures in the data set to show how the amount of available bulk data can affect the results. In all three cases, the average RMSD drops immediately as *λ*_3_ increases from 0 and reaches a minimum value for some *λ*_3_ > 0. This result shows that including the proposed population prior improves the accuracy of chromatin reconstruction in simulated sparse single-cell Hi-C data, and the improvement is greater when more bulk data are available.

### 3.2. Real data

**FIG. 5.**
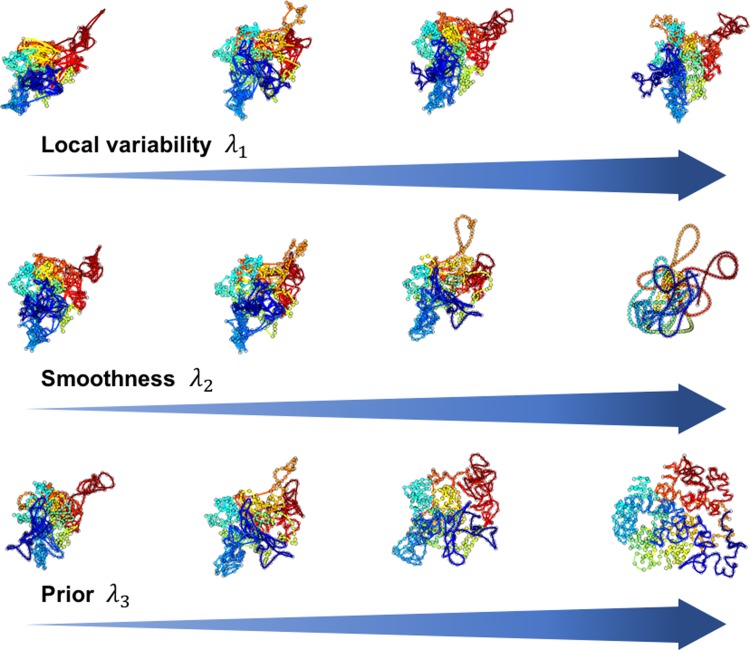
Effect of model parameters on 3D reconstruction quality. We compute the 3D reconstruction as a function of parameter values using the Hi-C data matrix associated with chromosome 19 in cell 1 of the mESC data set. The top row of structures from left to right shows the effect of an increased weight *λ*_1_ on the parameterization penalty *h*_1_. We vary *λ*_1_ = 0.01, 0.1, 1, 10 and fix *λ*_2_ = *λ*_3_ = 0 to obtain four solution curves with exponentially increasing penalty weight. The center row of structures from left to right shows the effect of an increasing weight *λ*_2_ on the smoothing penalty *h*_2_. Here we vary *λ*_2_ = 0.01, 0.1, 1, 10 and fix *λ*_1_ = 0.5 and *λ*_3_ = 0 to obtain these four solution curves. Finally, to show the effect of incorporating the bulk data—the mESC chromosome 19 population matrix—in the analysis, we vary *λ*_3_ = 0.01, 0.1, 1, 10 and fix *λ*_1_ = 0.5, *λ*_2_ = 1 to obtain the four structures on the bottom row. We computed all structures using the multiscale approach with *n* = 73, 146, 292, 584. 3D, three-dimensional; mESC, mouse embryonic stem cell.

We applied SIMBA3D to the reconstruction of chromosome structures from single-cell Hi-C data from mESC (Stevens et al., 2017). To highlight the influence of parameter selection on the results, Figure 5 illustrates the effect of the relative values of the three weights—*λ*_1_, *λ*_2_, and *λ*_3_—on the resulting estimated structures. As expected, higher values of these weights lead to increases in the respective properties they emphasize. For instance, an increase in *λ*_3_ leads to the chromosome structure bearing more resemblance to the structure estimated from the bulk data alone.

**FIG. 6.**
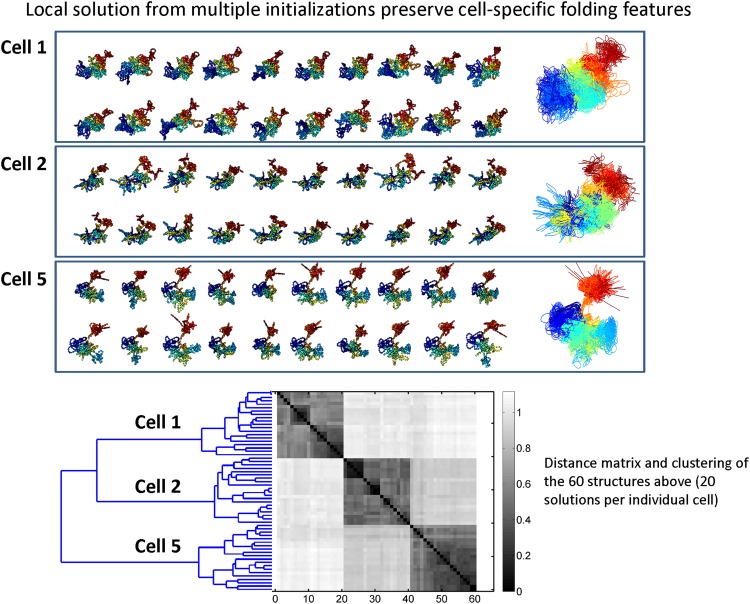
Similarity between ensembles of solutions across cells. Here we show twenty local solutions obtained for chromosome 19 in each of three cells—cell 1, cell 2, and cell 5—in the mESC data set using fixed *λ* = (0.5, 1, 0.1). We computed all structures using the multiscale approach with *n* = 73, 146, 292, 584, and for each cell, we used the same twenty random initializations at the smallest scale. All displayed solutions are rotationally aligned. For each cell, we show the twenty obtained solutions separately, and in addition, to help visualize the variability inherent to the local solutions within cells, we plot the three groups of solutions on top of each other in three respective windows. We then show the clustering of all 60 solutions in shape space via a 60 × 60 pairwise RMSD matrix and associated dendrogram plot.

Figure 6 studies the nature of solutions resulting from different initializations on the same data. Due to the vast search space in which the structure estimation is performed as well as the nonconvexity of the objective function, the optimization procedure in SIMBA3D cannot ensure convergence to a *unique* global solution. Instead, the output structure represents one of many different local optima that can be reached depending on the initialization. Despite the existence of several local optima, multiple configurations resulting from the same cells do in fact cluster together in the shape space, as illustrated using a pairwise RMSD matrix and dendrogram in this figure. The clustering observed here lends further validity to the inferred structures.

The use of the multiscale optimization technique is beneficial for several reasons. It first estimates broader, coarser structures and then adds smaller details, thereby avoiding the abundance of local traps present at the highest resolution. In addition to reaching a lower energy solution on average, it also speeds the algorithm significantly due to low-dimensional searches in the early stages. Figure 6 quantifies gains in computational cost and final energies due to this multiscale approach. An illustration of this method is shown in Supplementary Movie S1.

## 4. Conclusions

In conclusion, SIMBA3D is a Bayesian framework for estimating 3D chromosome structures from single-cell Hi-C data, using penalties for regularization of the estimated structures and using additional information from the bulk Hi-C data. Using multiscale optimization tools and a BFGS routine, it generates computationally efficient inferences and compares these across different initializations and different data (cells). Clustering of solutions in the shape space from the same cell data supports the validity of these solutions.

## Supplementary Material

Supplemental data

Supplemental data

Supplemental data

Supplemental data

Supplemental data
