# Supplementary material for: Bayesian Estimation of Three-Dimensional Chromosomal Structure from Single-Cell Hi-C Data
Source: J Comput Biol. 2019 Nov 7;26(11):1191–202. doi: 10.1089/cmb.2019.0100 (PMC6856950; doi:10.1089/cmb.2019.0100)
Supplement: Supplemental data [file Supp_Data.pdf]

Supplementary Text for: Bayesian Estimation of 3D Chromosomal Structure from Single Cell Hi-C Data

1 Gradient Expressions

For each term in the energy function E defined in Eq. 10 of the main paper, we compute the expression for the partial derivative at $x_i \in \mathbb{R}^3$ for each $i = 1, \ldots, n$. The ith partial derivative evaluated at X is a vector in $\mathbb{R}^3$, and thus, the full gradient evaluated at X is a member of $\mathbb{R}^{n \times 3}$. The ith partial derivative of g is given by

$$\frac{\partial}{\partial x_i} g(X|C, a, b) = -a \sum_{j \neq i} \frac{x_i - x_j}{\|x_i - x_j\|^2} (c_{ij} - b\|x_i - x_j\|^a) \ . \tag{1}$$

Before computing the partial derivatives of h_1 and h_2 it is useful to define $d_i = x_{i+1} - x_i$ and $w_i = \|d_i\|$ for $i = 1, \ldots, n-1$. Also, let $L = \sum_{i=1}^{n-1} w_i$ and $S = \sum_{i=1}^{n-1} w_i^2$. Now, the ith partial of h_1 for $i = 2, \ldots, n-1$ can be written as

$$\frac{\partial h_1}{\partial x_i} = d_{i-1} \left(\frac{2}{L^2} - \frac{2S}{L^3 w_{i-1}} \right) - d_i \left(\frac{2}{L^2} - \frac{2S}{L^3 w_i} \right) \ . \tag{2}$$

The remaining endpoint partial derivatives for h_1 are given as

$$\begin{aligned} \frac{\partial}{\partial x_1} h_1(X) &= -d_1 \left(\frac{2}{L^2} - \frac{2S}{L^3 w_1} \right) \\ \frac{\partial}{\partial x_n} h_1(X) &= d_{n-1} \left(\frac{2}{L^2} - \frac{2S}{L^3 w_{n-1}} \right), \end{aligned} \tag{3}$$

The ith partial derivative for h_2 is a bit trickier to compute than that of g and h_1 since it requires multiple applications of the chain rule, product rule, and quotient rule; nevertheless, the resulting expressions are still manageable. For $i = 3, \ldots, n-2$, we compute the ith partial as

$$\begin{aligned} \frac{\partial}{\partial x_i} h_2(X) = \frac{1}{n-2} \Bigg[&-d_{i-2} \left(\frac{1}{w_{i-2} w_{i-1}} \right) + d_{i-1} \left(\frac{d_{i-2} \cdot d_{i-1}}{w_{i-2} w_{i-1}^3} + \frac{1}{w_{i-1} w_i} + \frac{d_{i-1} \cdot d_i}{w_{i-1}^3 w_i} \right) \\ &- d_i \left(\frac{1}{w_{i-1} w_i} + \frac{d_{i-1} \cdot d_i}{w_{i-1} w_i^3} + \frac{d_i \cdot d_{i+1}}{w_i^3 w_{i+1}} \right) + d_{i+1} \left(\frac{1}{w_i w_{i+1}} \right) \Bigg] . \end{aligned} \tag{4}$$

The remaining four endpoint partial derivatives are as follows:

$$\begin{aligned} \frac{\partial}{\partial x_1} h_2(X) &= \frac{1}{n-2} \left[-d_1 \left(\frac{d_1 \cdot d_2}{w_1^3 w_2} \right) + d_2 \left(\frac{1}{w_1 w_2} \right) \right] \\ \frac{\partial}{\partial x_2} h_2(X) &= \frac{1}{n-2} \Bigg[d_1 \left(\frac{1}{w_1 w_2} + \frac{d_1 \cdot d_2}{w_1^3 w_2} \right) - d_2 \left(\frac{1}{w_1 w_2} + \frac{d_1 \cdot d_2}{w_1 w_2^3} + \frac{d_2 \cdot d_3}{w_2^3 w_3} \right) \\ &\quad + d_3 \left(\frac{1}{w_2 w_3} \right) \Bigg] \\ \frac{\partial}{\partial x_{n-1}} h_2(X) &= \frac{1}{n-2} \Bigg[-d_{n-3} \left(\frac{1}{w_{n-3} w_{n-2}} \right) + d_{n-2} \left(\frac{d_{n-3} \cdot d_{n-2}}{w_{n-3} w_{n-2}^3} + \frac{1}{w_{n-2} w_{n-1}} \right. \\ &\quad \left. + \frac{d_{n-2} \cdot d_{n-1}}{w_{n-2}^3 w_{n-1}} \right) - d_{n-1} \left(\frac{1}{w_{n-2} w_{n-1}} + \frac{d_{n-2} \cdot d_{n-1}}{w_{n-2} w_{n-1}^3} \right) \Bigg] \\ \frac{\partial}{\partial x_n} h_2(X) &= \frac{1}{n-2} \left[-d_{n-2} \left(\frac{1}{w_{n-2} w_{n-1}} \right) + d_{n-1} \left(\frac{d_{n-2} \cdot d_{n-1}}{w_{n-2} w_{n-1}^3} \right) \right] \ . \end{aligned} \tag{5}$$

Now, using Eqs. 1, 2, 3, 4, and 5, we can write the ith partial of E as

$$\frac{\partial}{\partial x_i}E(X|C,C',a,b,\lambda) = \frac{1}{M}\frac{\partial}{\partial x_i}g(X|\tilde{C},a,\tilde{b}) + \lambda_1\frac{\partial}{\partial x_i}h_1(X) + \lambda_2\frac{\partial}{\partial x_i}h_2(X) \quad (6)$$

and thus, using Eq. 6, we write the gradient of E in Eq. 11:

$$\nabla E(X|C,C',a,b,\lambda) = \begin{bmatrix} \frac{\partial E}{\partial x_1} \\ \vdots \\ \frac{\partial E}{\partial x_n} \end{bmatrix}. \quad (7)$$

2 Software Implementation and Optimization Techniques

Although most scientific computing platforms provide several built-in unconstrained optimization algorithms to solve such problems, we have selected Python as the programming language for SIMBA3D due to its ease of use across a broad scientific community. It is becoming widely adopted in the field because it is open source and does not require a proprietary license to run. Also, specifically addressing the problem at hand, Python's "scipy" package has a wealth of mature built-in scientific computing functions that are necessary for solving this type of optimization problem. In particular, the "scipy.optimize" module offers several popular options for unconstrained optimization algorithms, including Nelder-Mead, Powell's method, BFGS, Conjugate Gradient, etc. Python thus gives us the most flexibility and usability for the SIMBA3D code package.

After testing Python's built-in algorithms on various simulated and real datasets, we found the quasi-Newton methods BFGS and L-BFGS using our analytical gradient to yield the best performance in terms of numerical stability, computational efficiency, and solution quality when optimizing Eq. 10. With BFGS, the inverse Hessian operation is approximated recursively for each iteration, and for high dimensional optimizations (like for 3D chromosomal reconstruction) this procedure can become memory intensive. With the limited memory version L-BFGS, the approximated inverse Hessian operation depends on values stored from a fixed number of previous iterations. For details see Liu and Nocedal (1989), Byrd, Lu, et al. (1994), Nocedal and Wright (2006), and Gill, Murray, and Wright (1981). In our experience with applying these quasi-Newton methods specifically to genome architecture reconstruction, we found that BFGS more reliably finds solutions with slightly smaller energies, i.e. better quality solutions in terms of our objective function, than the L-BFGS. However, the reduction in computation time and memory offered by the the limited memory version makes it a competitive alternative.

In addition to Python's built-in optimization routines, we also tested our own implementation of Nesterov's accelerated gradient method Nesterov (1983). This method is a simple and elegant modification of the standard gradient descent that uses an additional "momentum" term to achieve the theoretically optimal convergence rate for a first order method. It has enjoyed a recent surge in popularity due to the publication of a series of physics-based convergence proofs Wibisono, Wilson, and Jordan (2016), Su, Boyd, and Candes (2015), and Wilson, Recht, and Jordan (2015) that are more widely relatable and understandable than Nesterov's original proofs. In many test cases we saw a significant computational speedup over the BFGS, and we see great promise in this optimization method in the future. Unfortunately, since the method is not a relaxation scheme, i.e. it does not necessarily decrease the objective function value after each iteration, we found it difficult to devise an appropriate

stopping criteria that would generalize to all data sets. Another difficulty that arose in our implementation of this algorithm was an automatic step size selection routine via backtracking, and thus achieving automated numerical stability for a general data set proved to be a challenge. We believe we can overcome these challenges to implementing this method, but in the interest of time and to remain within the scope of this research, we feel that BFGS performs well enough to use for SIMBA3D, especially when combined with the multiscale approach detailed in the main text.

3 Additional Results

Here we present a series of results that are not shown in the main text, which come from three computational experiments. The first experiment is carried out on simulated data and shows how the computation time increases as the number of nodes n increases. In the second experiment we execute an exhaustive parameter search by running the optimization with various settings of the penalty weights λ on Chromosome 19 of each of the eight single cells in the mESC data set. Finally, in the third experiment we compare the performance of the BFGS and L-BFGS with and without the multiscale approach in terms of computation times and final energies on all 20 chromosomes and all 8 cells in the data set. Fig. 4 in the main text summarizes the results of this experiment, but here we present the results in their entirety.

Table S1 and Fig. S1 summarize the results from the simulated computation time experiment. The ground truth curve is a unit length double spiral shape as seen in Fig. S1 (A). For each row of Table S1, the number of nodes in the double spiral curve was doubled using upsampling via spline interpolation, and a corresponding data matrix was generated from the upsampled curve. Fig. S1 (B) shows an example data matrix that corresponds with the curve and number of nodes presented in Fig. S1 (A). The entries of the upper triangular portion of each data matrix were simulated using the independent Poisson random variables $C_{ij} \sim Poisson(b\|x_i - x_j\|^a)$ with $a = -3$ and b set large enough to avoid simulating a sparse matrix. For each number of nodes n, ten initializations to the optimization procedure were generated as random samples of size n from the 3-dimensional standard multivariate normal distribution. Then for each number of nodes and for each initialization, the optimization was run using the BFGS without the multiscale approach, and the computation time and final energy were recorded in each case. However, in the case with 2560 nodes, the experiment was stopped prematurely after the sixth initialization due to an unreasonably long computation time. The penalty weight vector λ remained fixed for all optimizations in the experiment, with $\lambda_3 = 0$ due to the absence of simulated prior data. Fig. S1 (C) shows the evolution of the energy, i.e. the objective function evaluated at each iteration of the optimization algorithm, for one initialization and using the data given in panel (B), and panel (D) shows the final estimated solution curve. Panel (E) is a scatter plot of the log computation time using each of the ten initializations versus the log number of nodes, including a fitted regression line.

The results presented in Fig. 4 of the main text have been extended in Table S2 to include comparisons using BFGS and the limited memory version L-BFGS with and without the multiscale approach. The experiment shows how computation time varies with respect to the number of nodes by executing the optimization on all 20 chromosomes in each of the eight single cells. It also shows that one can use SIMBA3D on a standard laptop to achieve a full genome reconstruction within a practical time frame. The penalty weight vector λ was fixed throughout the experiment and set to have reasonable non-zero component values according to the results gathered from the previous experiment. Table S2 lists and plots the final computation times and energies from these reports along with other information describing the data.

Figure S1. Example of 3D shape reconstruction in simulated data. Computation time experiment on simulated data. (A) Ground truth curve using 80 points. (B) Simulated matrix using the Poisson model from Eq. 3. (C) Example of the energy evolution from a randomly initialized curve. (D) Example of a reconstructed curve from the data matrix. (E) Log scale computation time versus the log scale number of nodes plotted for each of the ten initializations.

Table S1. Computation time summary statistics on simulated data The mean and variance of the computation times for each number of nodes are computed after executing the optimization using ten different initializations. An example of one run for this experiment is shown in Fig. S1 for the case when there are 80 nodes.

Table S2. A Comparison of computation times and energies on the full chromosome data. There are 8 sheets – one for each of the 8 cells – and on each sheet there are 20 rows of data corresponding to the 20 chromosomes. The first 5 columns specify generic information about the single cell data matrix. The following columns correspond to computation times and final energies for single initialized runs using four different methods. A BFGS and L-BFGS label respectively indicates the use of the quasi-Newton BFGS or its limited memory version. The FS tag on the label indicates that the algorithm was initialized on the full scale space. For example with Cell 1 Chromosome 1, the BFGS FS column indicates that BFGS was used and the solution was initialized with a random sample of size 1923 from the standard multivariate normal distribution in $\mathbb{R}^3$. The MS tag on the label indicates that the curve was estimated using the multiscale approach detailed in the main text. In the multiscale approach the scale was roughly halved a total of four times; thus, for each chromosome the number of nodes in the initialization for the MS case is roughly $1/2^4$ that of the FS case.

Movie S1. Time-course optimization for chromosome 19. This movie displays the intermediate structures obtained during the optimization procedure. Rotating chromosome structure (top left). Stationary view of chromosome structure (top right). Energy as a function of iteration step (bottom left). Single cell contact matrix at the current resolution (bottom center). Bulk contact matrix at the current resolution (bottom right).

References

Byrd, Richard H., Peihuang Lu, et al. (1994). “A Limited-Memory Algorithm for Bound Constrained Optimization”. In: *SIAM JOURNAL ON SCIENTIFIC COMPUTING* 16, pp. 1190–1208.

Gill, P.E., W. Murray, and M.H. Wright (1981). *Practical optimization*. Academic Press. ISBN: 9780122839504. URL: `https://books.google.com/books?id=xUzvAAAAMAAJ`.

Liu, D. C. and J. Nocedal (Dec. 1989). “On the Limited Memory BFGS Method for Large Scale Optimization”. In: *Math. Program.* 45.3, pp. 503–528. ISSN: 0025-5610. DOI: `10.1007/BF01589116`. URL: `http://dx.doi.org/10.1007/BF01589116`.

Nesterov, Y. (1983). “A Method for Solving a Convex Programming Problem with Convergence Rate O(1/K2)”. In: *Soviet Mathematics Doklady* 27, pp. 372–367.

Nocedal, Jorge and Stephen J. Wright (2006). *Numerical Optimization*. Second. Springer Series in Operations Research and Financial Engineering. Springer New York. ISBN: 978-0-387-30303-1. DOI: `10.1007/978-0-387-40065-5`. URL: `http://dx.doi.org/10.1007/978-0-387-40065-5`.

Su, Weijie, Stephen Boyd, and Emmanuel J. Candes (2015). "A Differential Equation for Modeling Nesterov's Accelerated Gradient Method: Theory and Insights". In: eprint: arXiv:1503.01243v2.

Wibisono, Andre, Ashia C. Wilson, and Michael I. Jordan (2016). "A variational perspective on accelerated methods in optimization". In: *Proceedings of the National Academy of Sciences* 113.47, E7351–E7358. ISSN: 0027-8424. DOI: 10.1073/pnas.1614734113. eprint: http://www.pnas.org/content/113/47/E7351.full.pdf. URL: http://www.pnas.org/content/113/47/E7351.

Wilson, Ashia C., Benjamin Recht, and Michael I. Jordan (2015). "A Lyapunov Analysis of Momentum Methods in Optimization". In: eprint: arXiv:1611.02635v3.
